# STAT2 act a prognostic biomarker and associated with immune infiltration in kidney renal clear cell carcinoma

**DOI:** 10.1097/MD.0000000000033662

**Published:** 2023-04-28

**Authors:** Tao Zeng, Jianzhong Ye, Heng Wang, Wen Tian

**Affiliations:** a College of Medicine, Jingchu University of Technology, Jingmen, China; b College of Electronic Information Engineering, Jingchu University of Technology, Jingmen, China; c College of Computer Engineering, Jingchu University of Technology, Jingmen, China.

**Keywords:** bioinformatics analysis, immune checkpoint inhibitor, renal clear cell carcinoma, STAT2

## Abstract

Renal clear cell carcinoma (KIRC) is a malignancy of the renal epithelial cells with poor prognosis. Notably, the JAK-STAT pathway mediates cell proliferation and immune response. Accumulating evidence suggests that STATs act as immune checkpoint inhibitors in various cancers. Nonetheless, the role of STAT2 in KIRC remains elusive. Herein, analyses were performed using a series of interactive web databases including Oncomine, GEPIA and TIMER. In sub-group analyses, STAT2 was upregulated at both the mRNA and protein levels in KIRC patients. Besides, KIRC patients with high STAT2 expression exhibited a poor overall survival. Moreover, Cox regression analysis revealed that STAT2 expression, nodal metastasis and clinical stage were independent factors affecting the prognosis of KIRC patients. There was a significant positive correlation between STAT2 expression, and the abundance of immune cells as well as the expression of immune biomarker sets. In addition, STAT2 was found to be implicated in immune response, cytokine-cytokine receptor interaction, and Toll-like receptor signaling pathways. Also, several cancer-related kinases, miRNAs, and transcription factors associated with STAT2 were identified. Conclusively, we revealed that STAT2 is a potential prognosis biomarker and associated with immune infiltration in kidney renal clear cell carcinoma. This study offers additional data that will help in further research on the roles of STAT2 protein in carcinogenesis.

## 1. Introduction

Renal clear cell carcinoma (KIRC) is a malignancy of the renal epithelial cells. It accounts for up to 80% to 90% of renal cell carcinomas. Annually, at least 209,000 people are diagnosed with KIRC across the globe. Moreover, more than 102,000 patients annually succumb to the disease.^[1]^ Whist acknowledging the advances made in ultrasound and computed tomography for KIRC detection, 1/3 of patients are diagnosed at an advanced stage of the disease. This phenomenon ascribes to a lack of precise clinical symptoms of the disease.^[1]^ The prognosis of KIRC patients is poor particularly with patients in the advanced stage of the disease. Besides, less than 10% of KIRC patients in stage IV have a 5-year disease-specific survival. Despite checkpoint inhibitor-based immunotherapy becoming a superior therapeutic strategy for advanced malignancies, only a few immune checkpoint inhibitors have been identified for KIRC.^[2]^ Therefore, it is important to explore and authenticate precise and sensitive immune checkpoint inhibitors for KIRC immunotherapy.

The Janus kinase-signal transducers and activators of transcription (JAK-STAT) pathway regulates cytokine associated signaling by mediating cell proliferation, survival, differentiation, and immune response, which could influence tumorigenesis.^[3,4]^ The human genome codes for 7 STAT (STAT1/2/3/4/5A/5B/6) proteins. They function as transcriptional inducers, gene expression regulators via epigenetic modifications, and epithelial-mesenchymal transition mediators. Consequently, their functions generate a pro-tumorigenic microenvironment thereby promoting self-renewal and differentiation of cancer stem cells as well as establishing the formation of pre-metastatic niche.^[5,6]^ Accumulating evidence has demonstrated that inhibition of the JAK-STAT pathway is a potential target for drug development and immunotherapy in various cancers. In oral cancer, STAT2 is associated with activated T-Cell signature and better outcome.^[7]^ Moreover, high expression of STAT2 predicted poor prognosis in non-small cell lung cancer.^[8]^ STAT2 acted as a prognostic biomarker in colorectal cancer.^[9]^

We sought to explore the role of STAT2 in genomic alterations, its prognostic value, its associated biological functions, and its potential role in immune response. This was executed using multi-dimensional analysis tools of various public databases. As a result, our findings provide additional data for STAT2 and JAK-STAT pathway as potential therapeutic targets and treatment options for KIRC.

## 2. Materials and methods

### 2.1. ONCOMINE

Oncomine (www.oncomine.org) is a versatile systematic platform comprising 715 datasets and 86,733 samples. It was established for drug development and clinical practice.^[10]^ STAT2 levels in KIRC patients were compared to those of renal tissues of healthy persons by Oncomine analytic tools. A *P* value of .05 and a fold change of 1.5 were used as the thresholds. Then, the Student *t* test was used to evaluate the difference. This analysis was performed on October 30, 2019.

### 2.2. TIMER

TIMER (www.cistrome.shinyapps.io/timer/) is a comprehensive resource used for the clinical relevance of tumor-immune infiltrations.^[11]^ STAT2 levels in different types of tumors were explored using the “Diff Exp” module. Also, the levels of STAT2 in KIRC and its correlation with the abundance of immune cell infiltrates were explored using the “Gene” module. Additionally, the “correlation” module was used to analyze the correlation between STAT2 level and gene markers of immune cells. The gene markers and tumor-infiltrating immune cells, including CD8+ T cells, T cells (general), B cells, monocytes, TAMs, M1macrophages, M2 macrophages, neutrophils, natural killer cells, dendritic cells (DCs), T-helper 1 (Th1) cells, T-helper 2 (Th2) cells, follicular helper T (Tfh) cells, T-helper 17 (Th17) cells, Tregs, and exhausted T cells had been reported in previous studies.^[12–14]^ In the “survival” module, a Cox proportional hazard model was constructed to further identify the independent factors among immune cells influencing the prognosis of KIRC patients. These analyses were performed on October 30, 2019, while the cox proportional hazard model was constructed on August 26, 2020. These analyses were performed using The Cancer Genome Atlas (TCGA) KIRC dataset (n = 538).

### 2.3. UALCAN

UALCAN (www.ualcan.path.uab.edu/analysis.html) is a portal for promoting gene expression in cancer subgroups.^[15]^ The TCGA analysis module and the Clinical Proteome Tumor Analysis Consortium analysis module were used to determine the mRNA and protein levels of STAT2 respectively. This was repeated for the different KIRC sub-groups. The Student *t* test was also performed in this portal. *P* values less than .05 (*P* < .05) indicated that there were significant differences between the sub-groups. These analyses were performed using the TCGA KIRC dataset (n = 538) on October 30, 2019.

### 2.4. Prognosis analysis

The prognosis value of immune checkpoints in KIRC was analyzed with the Kaplan–Meier survival analysis. The median of STAT2 expression was used to divide KIRC patients into high and low expression groups. A log-rank test was used to evaluated the *P* values and hazard ratio (HR) with 95% confidence interval (CI).

### 2.5. cBioPortal

cBioPortal (www.cbioportal.org) is a portal used to explore, visualize and analyze multidimensional cancer genomics data.^[16]^ This tool was used to obtain the expression data of 538 TCGA KIRC samples (TCGA firehose legacy). mRNA expression z scores (RNA Seq V2 RSEM) were obtained using a z score threshold of ± 2.0. Protein expression z scores (RPPA) were obtained using a z score threshold of ± 2.0. Also, genetic alterations associated with STAT2 expression, the prognostic significance of STAT2, and the neighboring gene networks associated with STAT2 were analyzed.

### 2.6. DAVID 6.8

DAVID 6.8 (www.david.ncifcrf.gov/home.jsp) is a functional annotation tool used to identify enriched biological themes.^[17]^ The extracted neighboring genes related to STAT2 expression were submitted to DAVID 6.8 tool for Gene Ontology (GO) and Kyoto Encyclopedia of Genes and Genomes (KEGG) pathway analysis. Then, the R program was used to visualize the results using the “clusterProfiler” package. This analysis was performed on October 30, 2019.

### 2.7. LinkedOmics

LinkedOmics (www.linkedomics.org) is a systematic platform used to explore and analyze cancer-related multi-omics data.^[18]^ The Pearson’s Correlation test was performed based on the data of TCGA KIRC samples. STAT2 associated genes were explored using the “LinkFinder” module. Subsequently, the “LinkInterpreter” module was used to transform the STAT2 and associated genes data into biologically comprehensible data through pathway and network analyses. The pathways and network analyses included GO, KEGG pathways, kinase-target, miRNA-target enrichment, and transcription factor-target network. The minimum number of genes (size), the simulation value, and the *P* value cutoffs were set at 3, 500, and .05 respectively. These analyses were performed using the TCGA KIRC dataset (n = 538) on October 30, 2019.

### 2.8. Open targets

Open targets (www.targetvalidation.org) is a comprehensive portal for identifying diseases associated with a specific target or potential drug targets associated with a disease.^[19]^ Herein, this tool was used to identify diseases associated with STAT2. This analysis was performed on October 30, 2019.

### 2.9. GeneMANIA

GeneMANIA (www.genemania.org) is a protein-protein interaction (PPI) network constructing a portal that helps in understanding the underlying functions of target genes.^[20]^ Kinase_LYN network, MIR-337 network, and ETS transcription factor network genes were submitted to GeneMANIA geared towards visualizing them and understanding their functions. This analysis was performed on October 30, 2019.

## 3. Results

### 3.1. STAT2 expression in KIRC

**Figure 1.**
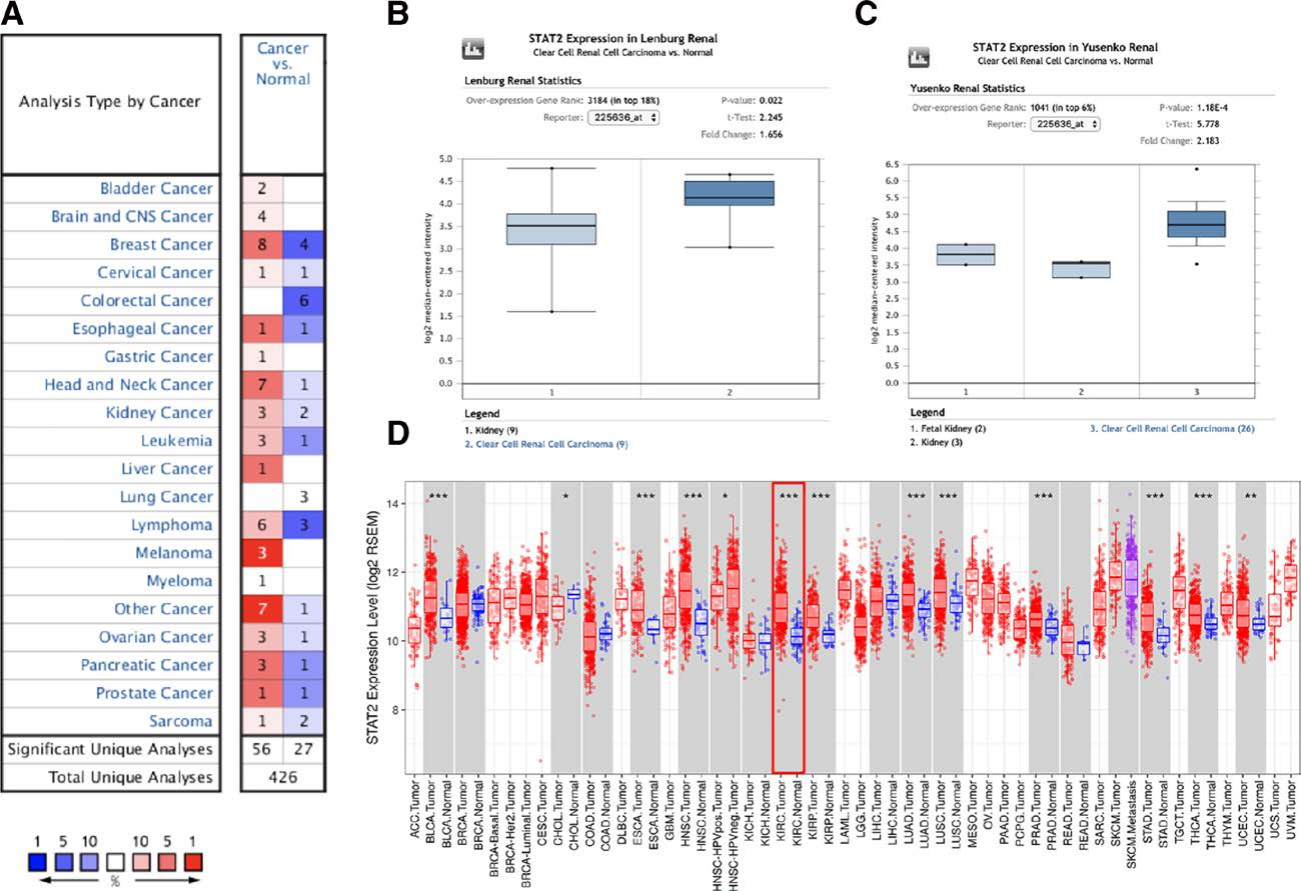
STAT2 expression in KIRC. (A) upregulation or downregulation of STAT2 in data sets of different cancers compared with normal tissues (Oncomine). (B–C) Box plot about STAT2 expression in Lenburg and Yusenko KIRC dataset (Oncomine). (D) upregulation or downregulation of STAT2 expression in different type of malignancies (TIMER) *P < .05, **P < .01, ***P < .001. KIRC = renal clear cell carcinoma.

STAT2 mRNA and protein expression in KIRC was detected. In the Oncomine database, significant upregulation of *STAT2* mRNA expression in KIRC tissues was observed (*P* < .05) (Fig. 1A). Based on Lenburg’s dataset, upregulation of *STAT2* mRNA expression was observed in KIRC tissues (fold change = 1.656, *P* value = .022) (Fig. 1B). Yusenko et al reported the overexpression of *STAT2* mRNA expression in KIRC tissues (fold change = 2.183, *P* value = 1.18E-04) (Fig. 1C). In the same line, data from TIMER revealed a significant upregulation in *STAT2* mRNA levels in KIRC patients (*P* < .001) (Fig. 1D).

**Figure 2.**
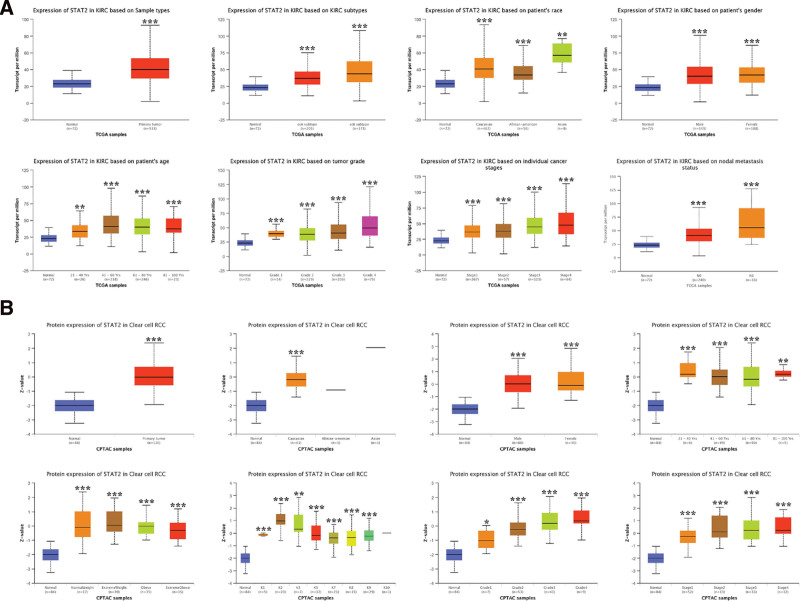
The association between STAT2 expression and clinicopathologic feature. (A) STAT2 mRNA expression in subgroups of patients with KIRC based on sample types, race, gender, age, weight, tumor grade, cancer stage, and nodal metastatic status (UALCAN). (B) STAT2 protein expression in subgroups of patients with KIRC based on sample types, race, gender, age, weight, tumor grade, cancer stage, and nodal metastatic status (UALCAN). *P < .05; **P < .01; ***P < .001. CPTAC = Clinical Proteome Tumor Analysis Consortium, KIRC = renal clear cell carcinoma, RCC = renal cell carcinoma.

Further, the mRNA and protein expression of STAT2 and their association with the clinicopathologic features of KIRC was evaluated. As a result, the upregulation in expression of *STAT2* mRNA was observed in KIRC patients compared to the normal healthy persons in the sub-group analyses based on sample types, race, gender, age, KIRC subtypes, tumor grade, cancer stages, and nodal metastasis status (Fig. 2A).

Similarly, KIRC patients exhibited a significant upregulation in the expression of STAT2 protein compared to the normal healthy persons in the sub-group analyses based on sample types, race, gender, age, weight, tumor grade, cancer stages, and nodal metastasis status (Fig. 2B). These findings demonstrated STAT2 as a potential diagnostic biomarker for KIRC.

### 3.2. The prognostic value of STAT2 in KIRC

**Figure 3.**
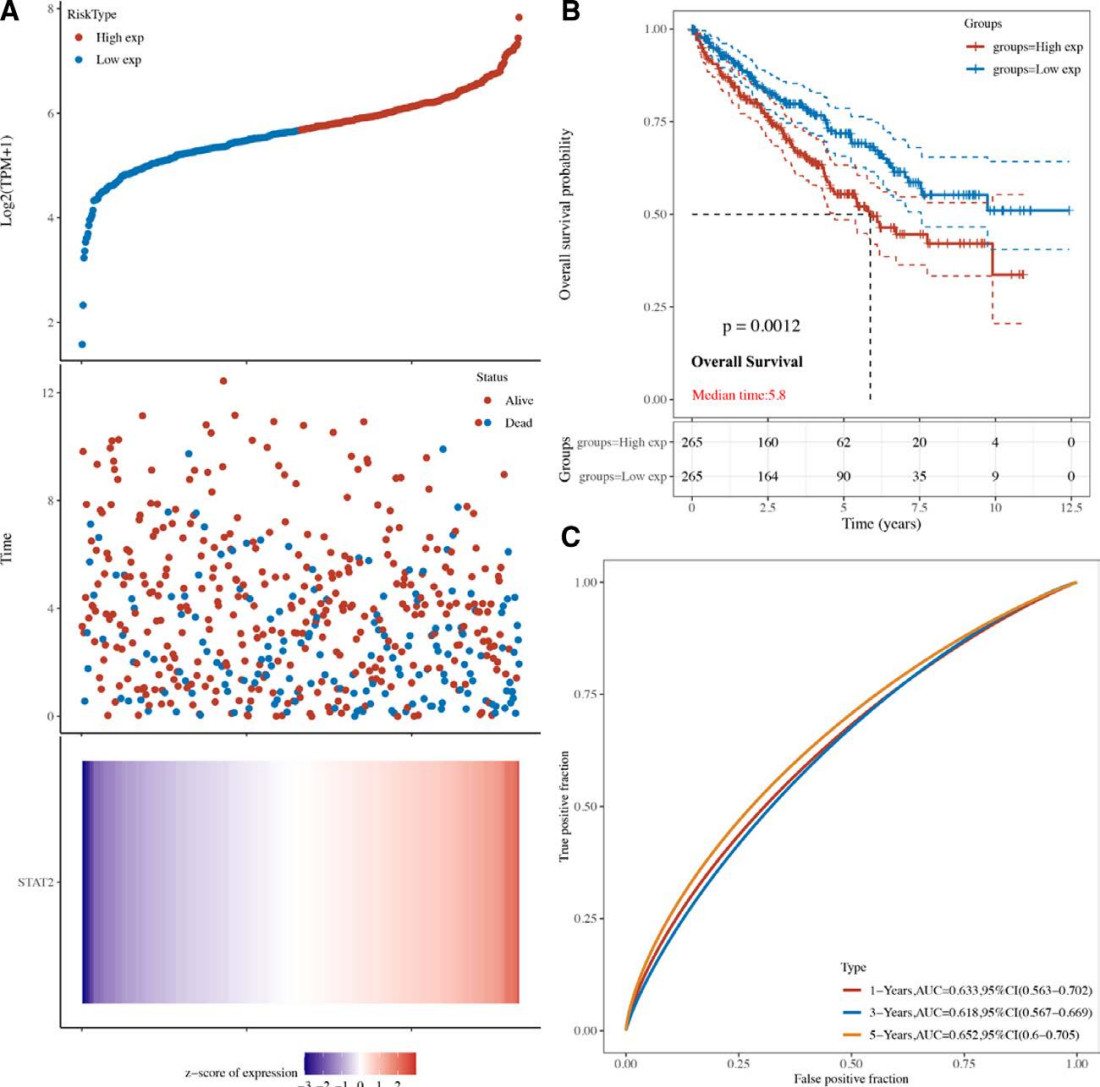
The overall survival analysis of STAT2 in KIRC. (A) The risk score, survival status and gene expression of each patients. (B) Kaplan–Meier overall survival curve of STAT2 in KIRC patients with high and low STAT2 expression. (C) Time-dependent ROC of STAT2 in predicting the prognosis of KIRC patients. KIRC = renal clear cell carcinoma, ROC = receiver operating characteristic.

**Figure 4.**
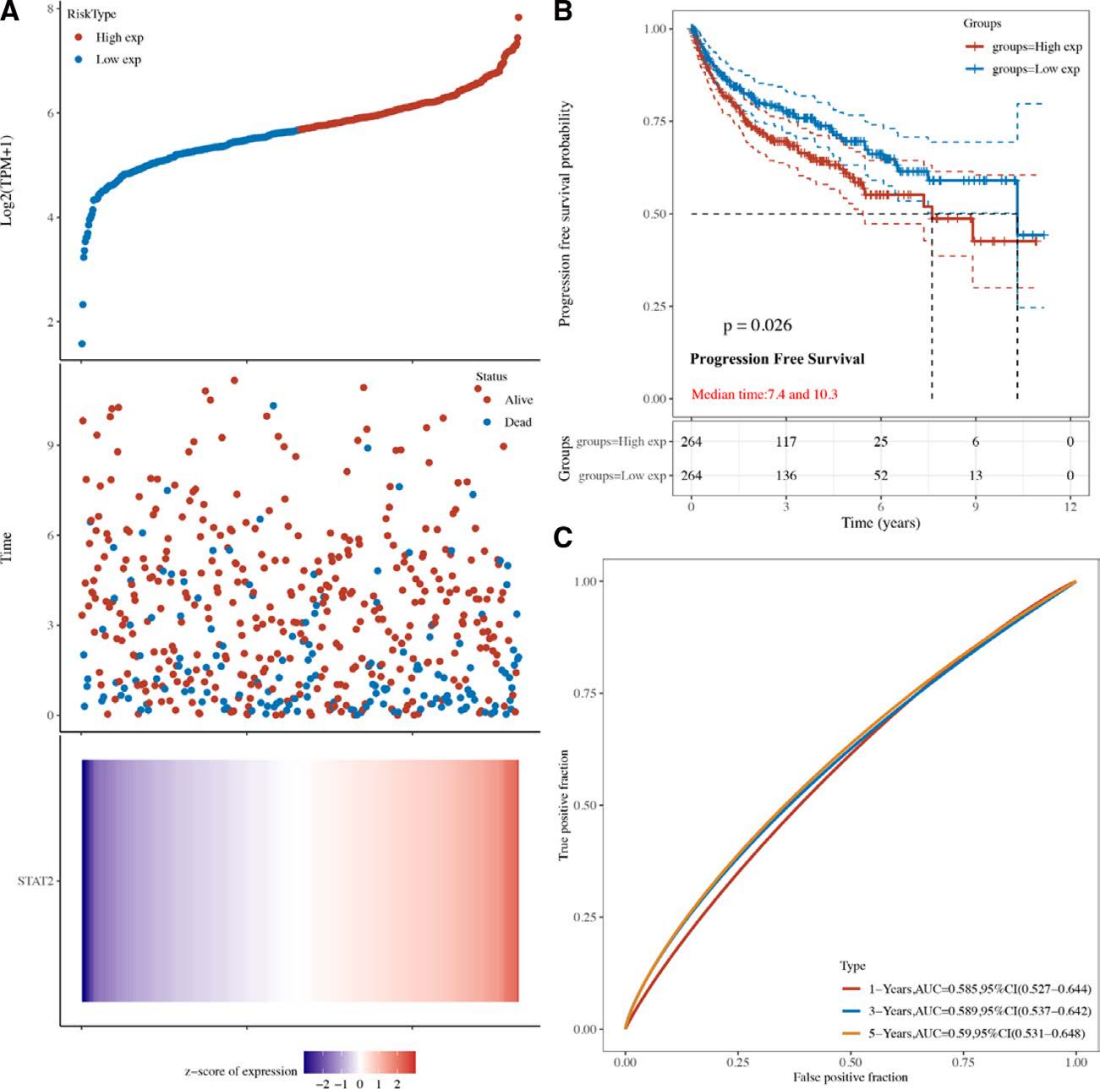
The progression free survival analysis of STAT2 in KIRC. (A) The risk score, survival status and gene expression of each patients. (B) Kaplan–Meier progression free survival curve of STAT2 in KIRC patients with high and low STAT2 expression. (C) Time-dependent ROC of STAT2 in predicting the prognosis of KIRC patients. KIRC = renal clear cell carcinoma, ROC = receiver operating characteristic.

**Figure 5.**
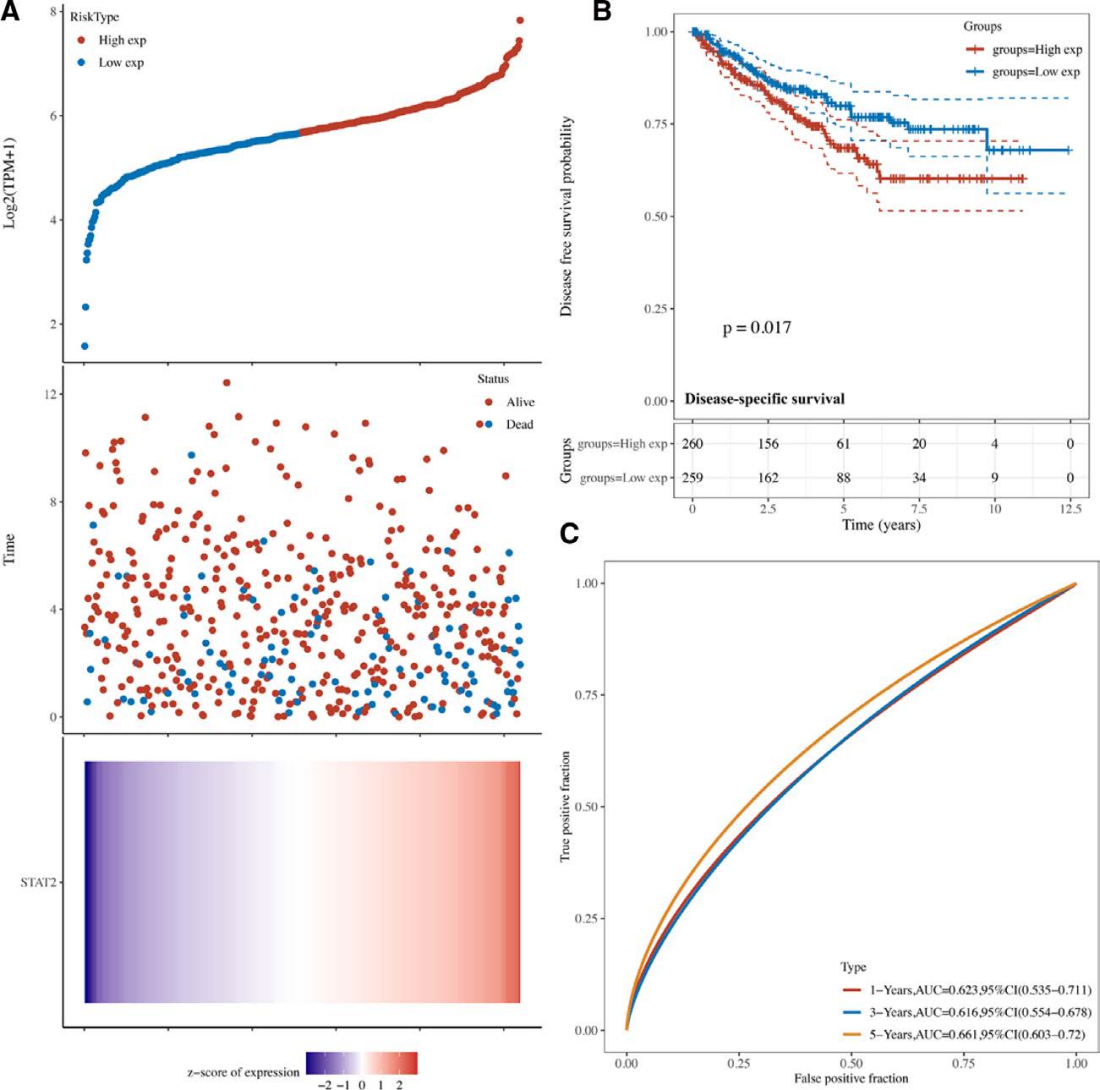
The disease-specific survival analysis of STAT2 in KIRC. (A) The risk score, survival status and gene expression of each patients. (B) Kaplan–Meier disease-specific survival curve of STAT2 in KIRC patients with high and low STAT2 expression. (C) Time-dependent ROC of STAT2 in predicting the prognosis of KIRC patients. KIRC = renal clear cell carcinoma, ROC = receiver operating characteristic.

Cox regression analysis was performed to calculate the risk score of STAT2 in KIRC and the Kaplan–Meier curves were drawn according to the risk score of each patient. The risk score of each patient in overall survival (OS), progression free Survival (PFS), and disease-specific survival (DFS) was shown in Figures 3A, 4A, and 5A. STAD patients with high expression of STAT2 expression had a poor OS (*P* = .0012, Fig. 3B), PFS (*P* = .026, Fig. 4B), and DFS (*P* = .017, Fig. 5B). with 5-year AUC of 0.652 (Fig. 3C), 0.59 (Fig. 4C), and 0.661(Fig. 5C), respectively. These results indicated that STAT2 served as potential prognostic biomarkers in KIRC.

### 3.3. The correlation between STAT2 expression and immune biomarker sets in KIRC

**Figure 6.**
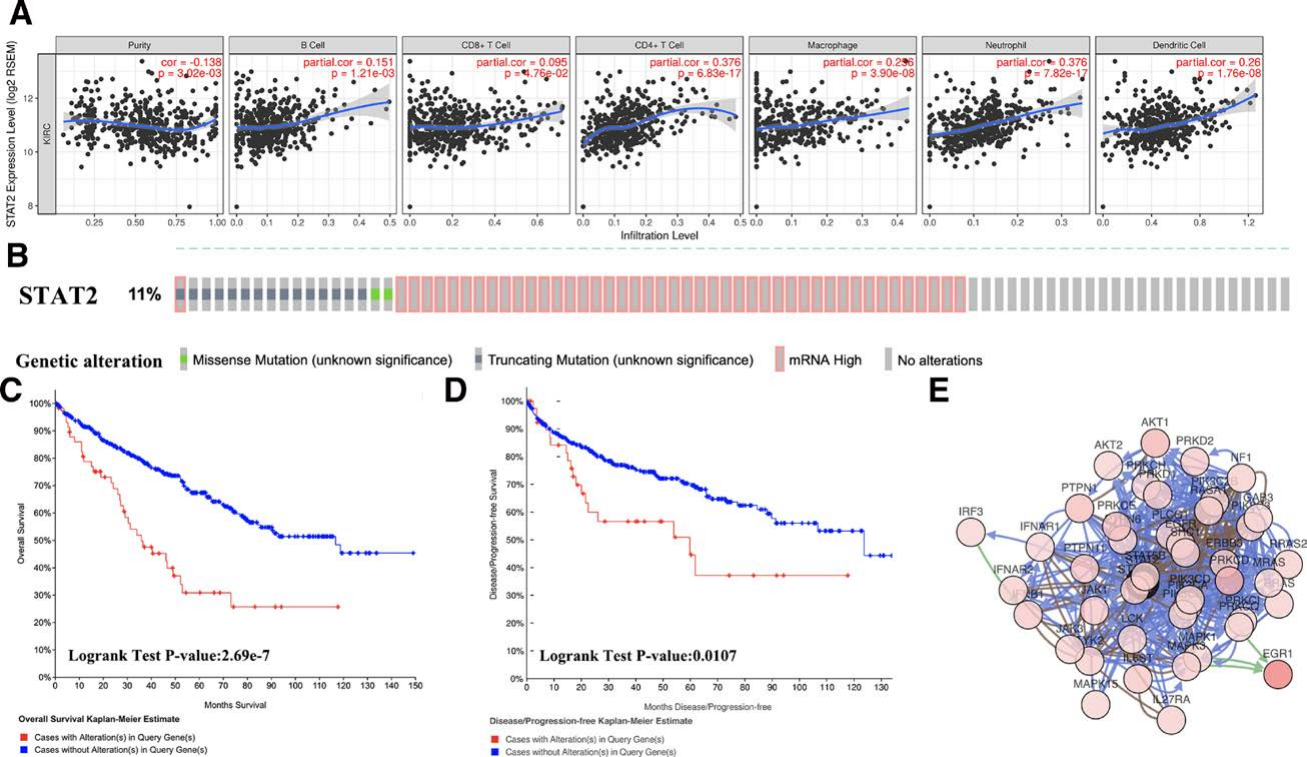
Immune infiltration, genetic alteration, survival and gene-gene interaction network of STAT2 in KIRC patients. (A) Correlation analysis of STAT2 and different immune cell level in KIRC (TIMER). (B) STAT2 genetic alteration in KIRC. The OncoPrint provides an overview of genomic alterations in STAT2 affecting individual samples (columns) in KIRC from the TCGA. The different types of genetic alterations are highlighted in different colors. (C and D) The overall and disease-free survival curves in KIRC cases with/without STAT2 alterations. (E) gene-gene interaction network of STAT2 and the neighbor gene (genetic alteration > 10%) in KIRC. KIRC = renal clear cell carcinoma.

Further, the correlation between *STAT2* expression and immune biomarker sets of KIRC was evaluated using TIMER. A significant positive correlation was found between *STAT2* expression and the abundance of B cells (Cor = 0.151, *P* = 1.21e-03), CD8+ T cells (Cor = 0.095, *P* = 4.76e-02), CD4+ T cells (Cor = 0.376, *P* = 6.83e-17), macrophages (Cor = 0.256, *P* = 3.90-08), neutrophils (Cor = 0.376, *P* = 7.82e-17), and dendritic cells (Cor = 0.26, *P* = 1.76e-08) in KIRC patients (Fig. 6A). A Cox proportional hazard model was constructed to further identify the independent factors among immune cells affecting the prognosis of KIRC patients. As a result, CD8+ T cells, CD4+ T cells, Macrophage, and STAT2 were revealed as the independent factors influencing the prognosis of KIRC patients (Table 1). The relationship between *STAT2* expression and immune biomarker sets in KIRC was further explored. *STAT2* expression was significantly positively correlated with the majority of the immune biomarker sets (Table 2, *P* < .05). Immune biomarkers of T cells (CD3D, CD3E, and CD2), B cells (CD19 and CD79A), monocytes (CD86 and CD11) M2 macrophages (CD163, VSIG4, and MS4A4A), and neutrophils (CD66b, CD11b, and CCR7) were positively correlated with STAT2 (Table 2, *P* < .05). Moreover, immune biomarkers of the CD8+ T cells (CD8A and CD8B) and Natural killer cells (KIR2DL4, KIR3DL2, and KIR3DL3) showed a positive weak correlation with STAT2 (Table 2, *P* < .05). Immune biomarkers of the dendritic cells showed a strong positive correlation with STAT2 except CD1C. Similarly, there was a significant positive correlation of immune biomarkers of Th1(T-bet, STAT4, STAT1, IFN-g, and TNF-a), Th2 (GATA3, STAT6, STAT5A, and IL13), Tfh (BCL6 and IL21), and Treg (FOXP3, CCR8, STAT5B, and TGFb) (Table 2, *P* < .05). Moreover, *STAT2* expression positively correlated with immune biomarkers of T cell exhaustion (PD-1, CTLA4, LAG3, and GZMB) (Table 2, *P* < .05). These results indicated that *STAT2* was highly significant in immune escape in the KIRC microenvironment. This demonstrated that *STAT2* is a potential immunotherapeutic target for KIRC therapy.

**Table 1:** Cox Proportional Hazard Model of STAT2 and immune cells in KIRC.

	Coef	HR	95% CI_l	95% CI_u	*P* value	Sig
B cells	−1.253	0.286	0.010	7.990	.461	
CD8+ T cells	−1.584	0.205	0.046	0.925	.039	*
CD4+ T cells	−3.503	0.030	0.001	0.642	.025	*
Macrophage	−3.067	0.047	0.004	0.491	.011	*
Neutrophil	0.131	1.140	0.017	76.332	.951	
Dendritic	1.121	3.068	0.493	19.073	.229	
STAT2	1.022	2.779	2.092	3.690	.000	***

Likelihood ratio test *P* = 1.66e-09; Wald test *P* = 2.58e-10; Score (Logrank) test *P* = 2.22e-09.

KIRC = renal clear cell carcinoma.

* *P* < .05.

*** *P* < .001.

**Table 2:** Correlation analysis between STAT2 and relate genes and markers of immune cells in KIRC (TIMER).

Description	Gene markers	KIRC			
		None		Purity	
		Cor	*P* value	Cor	*P* value
CD8+ T cell	CD8A	0.397	***	0.35	***
	CD8B	0.395	***	0.359	***
T cell (general)	CD3D	0.455	***	0.415	***
	CD3E	0.474	***	0.434	***
	CD2	0.484	***	0.441	***
B cell	CD19	0.437	***	0.402	***
	CD79A	0.391	***	036	***
Monocyte	CD86	0.43	***	0.418	***
	CD115 (CSF1R)	0.471	***	0.465	***
TAM	CCL2	−0.021	.625	−0.072	.124
	CD68	0.382	***	0.393	***
	IL10	0.396	***	0.381	***
M1 Macrophage	INOS (NOS2)	−0.159	***	−0.191	***
	IRF5	0.424	***	0.417	***
	COX2 (PTGS2)	0.11	*	0.067	.149
M2 Macrophage	CD163	0.302	***	0.309	***
	VSIG4	0.429	***	0.429	***
	MS4A4A	0.323	***	0.312	***
Neutrophils	CD66b (CEACAM8)	0.098	*	0.119	*
	CD11b (ITGAM)	0.482	***	0.47	***
	CCR7	0.37	***	0.356	***
Natural killer cell	KIR2DL1	0.052	.229	0.043	.354
	KIR2DL3	0.07	.106	0.186	.658
	KIR2DL4	0.277	***	0.277	***
	KIR3DL1	−0.006	.895	0.002	.971
	KIR3DL2	0.154	***	0.137	**
	KIR3DL3	0.125	**	0.108	*
	KIR2DS4	0.018	.674	0.009	.845
Dendritic cell	HLA-DPB1	0.342	***	0.328	***
	HLA-DQB1	0.22	***	0.182	***
	HLA-DRA	0.325	***	0.319	***
	HLA-DPA1	0.315	***	0.293	***
	BDCA-1 (CD1C)	0.11	*	0.087	.613
	BDCA-4 (NRP1)	−0.132	**	−0.159	***
	CD11c (ITGAX)	0.588	***	0.577	***
Th1	T-bet (TBX21)	0.375	***	0.349	***
	STAT4	0.504	***	0.471	***
	STAT1	0.304	***	0.331	***
	IFN-g (IFNG)	0.476	***	0.445	***
	TNF-a (TNF)	0.376	***	0.365	***
Th2	GATA3	0.23	***	0.213	***
	STAT6	0.376	***	0.4	***
	STAT5A	0.486	***	0.222	***
	IL13	0.318	***	0.302	***
Tfh	BCL6	0.268	***	0.276	***
	IL21	0.194	***	0.179	***
Th17	STAT3	0.083	*	0.061	.194
	IL17A	0.096	*	0.06	.200
Treg	FOXP3	0.565	***	0.336	***
	CCR8	0.464	***	0.439	***
	STAT5B	0.14	**	−0.138	**
	TGFb (TGFB1)	0.277	***	0.242	***
T cell exhaustion	PD-1 (PDCD1)	0.478	***	0.448	***
	CTLA4	0.549	***	0.52	***
	LAG3	0.523	***	0.477	***
	TIM-3 (HAVCR2)	0.045	.301	0.02	.674
	GZMB	0.259	***	0.223	***

None, Correlation analysis adjusted by nothing. Purity, Correlation analysis adjusted by tumor purity.

KIRC = kidney renal clear cell carcinoma.

* *P* < .05.

** *P* < .01.

*** *P* < .001.

### 3.4. Genetic alteration, survival, and neighbor gene biological interaction network analysis of STAT2 in KIRC

**Figure 7.**
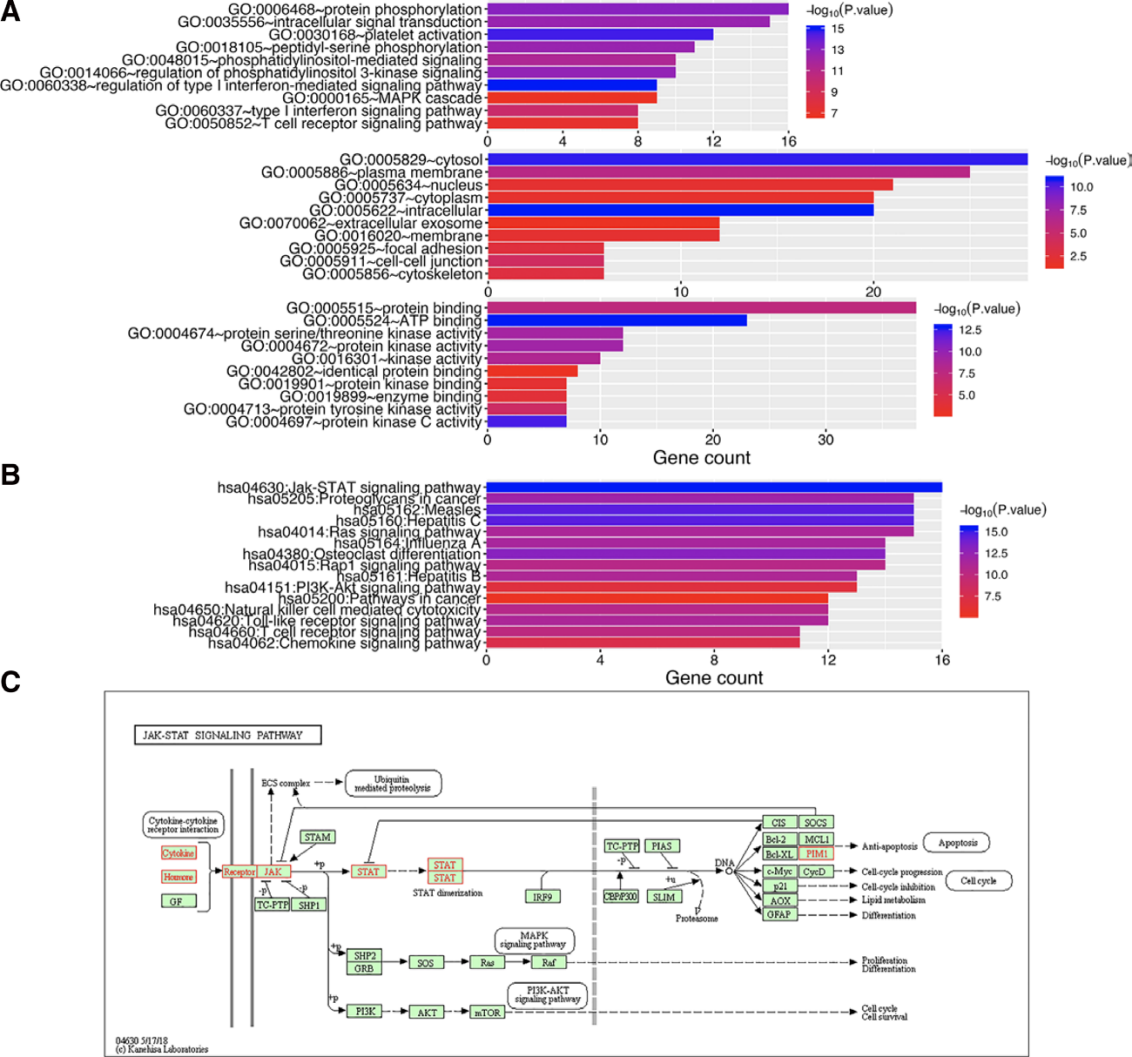
Functional analysis of STAT2 and neighboring genes in KIRC (DAVID). (A) GO enrichment analysis (B) KEGG pathway analysis. (C) KEGG pathway annotations of the JAK-STAT signaling pathway. GO = gene ontology, JAK-STAT = Janus kinase-signal transducers and activators of transcription, KEGG = Kyoto Encyclopedia of Genes and Genomes, KIRC = renal clear cell carcinoma.

Genetic alteration of STAT2 in KIRC patients was evaluated using cBioportal. In total, 66 out of the 538 (11%) KIRC patients exhibited STAT2 associated genetic alteration (Fig. 6B). STAT2 associated genetic alterations included mutations (16 cases representing 2.97%), mRNA upregulation (44 cases representing 8.18%), and multiple alterations (1 case representing 0.19%). The “survival” module of the cBioPortal revealed that KIRC patients with STAT2 alteration exhibited a poor overall survival (*P* = 2.69e-7) (Fig. 6C) and disease-free survival (*P* = .0107) (Fig. 6D) compared to KIRC patients without STAT2 alteration. Network construction of STAT2-neighboring genes revealed that there were 33 STAT2-neighboring genes altered at frequencies of more than 5%. EGR1 (20.8%) ranked top as the most frequent alteration (Fig. 6E; Table 3). Analysis of enriched GO terms of these genes demonstrated that these genes were primarily associated with signal transduction, focal adhesion, protein binding, and kinase activity (Fig. 7A). Moreover, the KEGG pathway analysis revealed that these genes were mainly associated with the JAK-STAT signaling pathway, proteoglycans in cancer, P13K-Akt signaling pathway, Ras signaling pathway, and chemokine signaling pathway (Fig. 7B and C).

**Table 3:** The type and frequency of STAT2 neighbor gene alterations in KIRC (cBioPortal).

Gene symbol	Amplification	Homozygous deletion	Up-regulation	Down-regulation	Mutation	Total alteration
AKT1	0	0.4	3.5	6.5	0.2	10.4
AKT2	0.2	0	4.5	0.9	0.6	5.9
EGFR	0	0.2	4.8	0	1.7	6.5
EGR1	13.6	0	7.2	0	1.7	20.8
ERBB3	0	0	5.2	0	1.1	6.3
GAB3	0.9	0.2	4.3	0	0.4	5.8
IFNAR1	0	0	2.6	3.0	0.4	5.8
IFNAR2	0	0	3.9	2.8	0	6.7
IFNB1	0	0.2	6.9	0	0.4	7.4
IL27RA	0	0	6.1	0	0.6	6.7
IL6ST	0	0	6.7	0	0.6	7.6
IRF3	0	0.2	5.4	0	0	5.6
JAK1	0	0.2	1.7	3.5	2.4	7.6
STAT2	0	0	5.4	0	0	5.9
LCK	0	0	6.3	0	0	6.3
MAPK1	0	0	4.1	1.5	0	5.6
MAPK15	0	0.7	5	0	0.2	5.9
MAPK3	0.2	0	4.3	2.4	0.2	6.9
MRAS	0.7	0.2	4.5	0	0.2	5.6
NF1	0.2	0	3.5	1.1	2	6.7
PIK3C2B	0.6	0	3.2	0.2	2.0	5.9
PIK3C3	0.2	0.4	3.2	3.2	0.4	6.3
PIK3CA	2.0	0	2.6	0.9	1.9	7.1
PIK3CB	0.7	0.2	3.3	0.6	1.3	6.1
PIK3CD	0	0.2	5.2	0.2	0.2	5.8
PLCG1	0	0	5.2	0.9	0.4	6.5
PRKCD	0	9.3	1.3	0	1.3	11.3
PRKCE	0	0.6	6.9	0.4	0.7	8.6
PRKCH	0.2	0.6	2.6	1.7	0.7	5.8
PRKCI	1.7	0	4.5	0	0.4	5.9
PRKCQ	0.2	0.2	4.6	0	0.7	5.6
PRKD1	0.2	0	3.2	3.3	0.6	7.1
PRKD2	0.2	0	4.5	2	0.7	7.4
PTPN1	0.4	0	8.6	0.2	0.2	9.1
PTPN11	0.2	0	5.9	1.7	0.2	8
PTPN6	0	0	5.4	0	0.6	5.9
RASA1	0.4	0	4.5	1.3	0.6	6.7
RRAS	0	0.2	6.1	0	0	6.3
RRAS2	0	0	5.2	0	1.5	6.7
SHAC1	0.6	0	5.2	0	0.6	6.3
STAT1	0.9	0.2	5.2	0	0.7	7.1
STAT5B	0.2	0	1.7	3.7	0.7	6.1
TYK2	0	0.2	4.8	0.7	1.1	6.9

KIRC = kidney renal clear cell carcinoma.

### 3.5. GO and KEGG pathway analyses of co-expression genes correlated with STAT2 in KIRC

**Figure 8.**
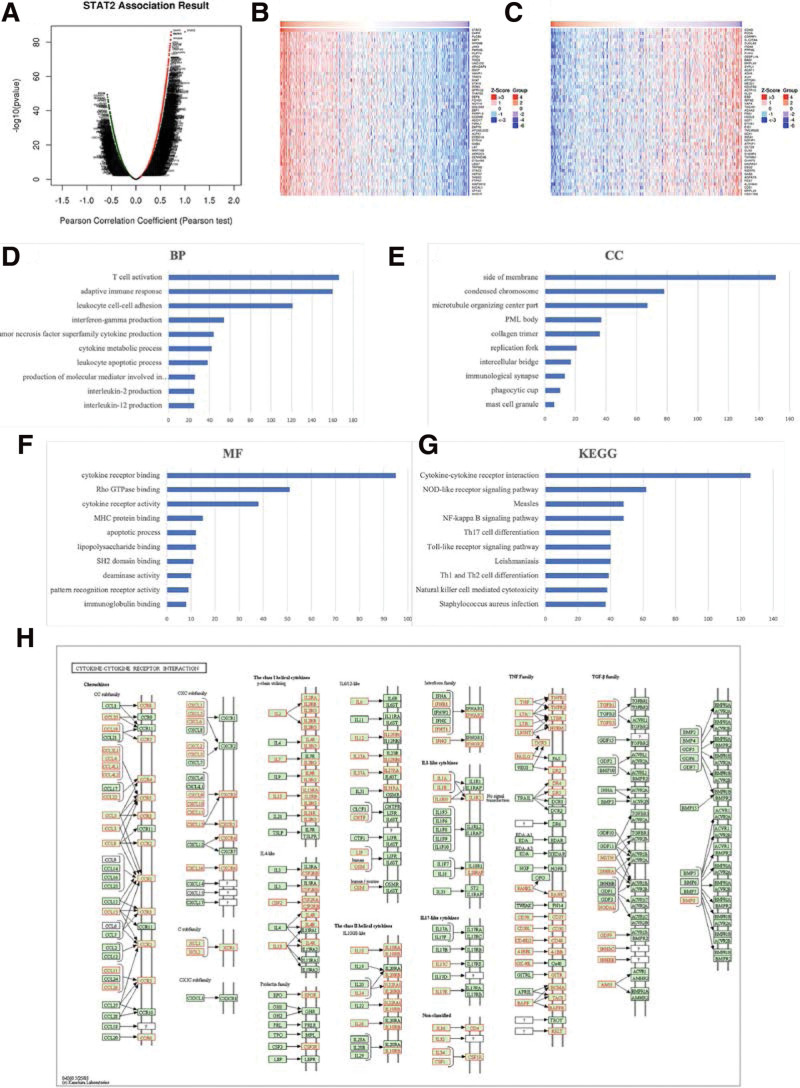
Associated genes, GO and KEGG pathway annotation analysis of STAT2 in KIRC (LinkedOmics). (A) A Pearson test was used to analyze correlations between STAT2 and genes differentially expressed in KIRC. (B) Heat maps of 50 genes most positively associated with STAT2 in KIRC. Red indicates positively correlated genes and green indicates negatively correlated genes. (C) Heat maps of 50 genes most negatively associated with STAT2 in KIRC. Red indicates positively correlated genes and green indicates negatively correlated genes (D) Biological process (BP) analysis. (E) Cellular Component (CC) analysis. (F) Molecular function (MF) analysis. (G) Kyoto Encyclopedia of Genes and Genomes (KEGG) pathway analysis. (H) KEGG pathway annotations of cytokine-cytokine receptor interaction. The significantly enriched GO annotations and KEGG pathways of STAT2 co-expression genes in KIRC were analyzed using GSEA. GO = gene ontology, KIRC = renal clear cell carcinoma.

**Figure 9.**
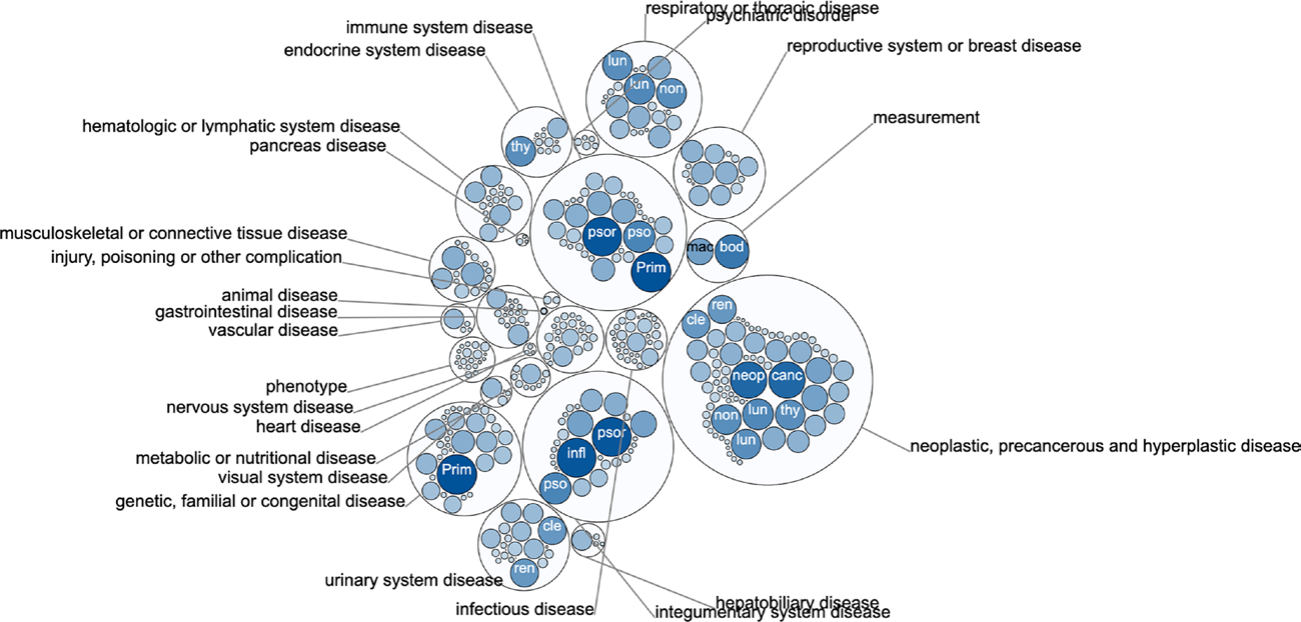
Bubbles shows the diseases associated with STAT2 (Open Targets).

This study used the transcriptional level data of 533 TCGA KIRC patients. A total of 9217 genes (dark red dots) showed a positive correlation with STAT2 whereas 4562 genes (dark green dots) showed a negative correlation with STAT2 (false discovery rate < 0.05) (Fig. 8A). A total of 50 genes most positively and negatively correlated with STAT2 were extracted and presented in a heat map (Fig. 8B and C). CHFR (cor = 0.721, *P* = 1.224e-86), XAF1 (cor = 0.7153, *P* = 1.12e-84), PLCB2 (cor = 0.7156, *P* = 8.579e-85), and MYO9B (cor = 0.7074, *P* = 4.725e-82) was ranked as the top 4 (Figure S1, Supplemental Digital Content, http://links.lww.com/MD/I904). GO enrichment function analyses revealed that the STAT2-associated genes encoded proteins were implicated in T cell activation, adaptive immune response, cytokine metabolic process, immunological synapse, cytokine receptor binding and activity, and apoptosis (Fig. 8D–F). In the same line, KEGG pathway analyses revealed that the STAT2-associated genes were enriched in cytokine-cytokine receptor interaction, NOD-like receptor signaling pathways, Toll-like receptor signaling pathway, and Th1 and Th2 cell differentiation (Fig. 8G and H). Moreover, the Open Targets portal revealed that STAT2 was associated with immune system disease and urinary system disease (Fig. 9). These findings indicate that STAT2 might be highly significant in immune escape in the KIRC microenvironment suggesting that STAT2 is a potential immune checkpoint inhibitor for KIRC therapy. However, further experiments are necessary to validate these findings.

### 3.6. Prognostic analysis of STAT2 in KIRC based on different immune cells

**Figure 10.**
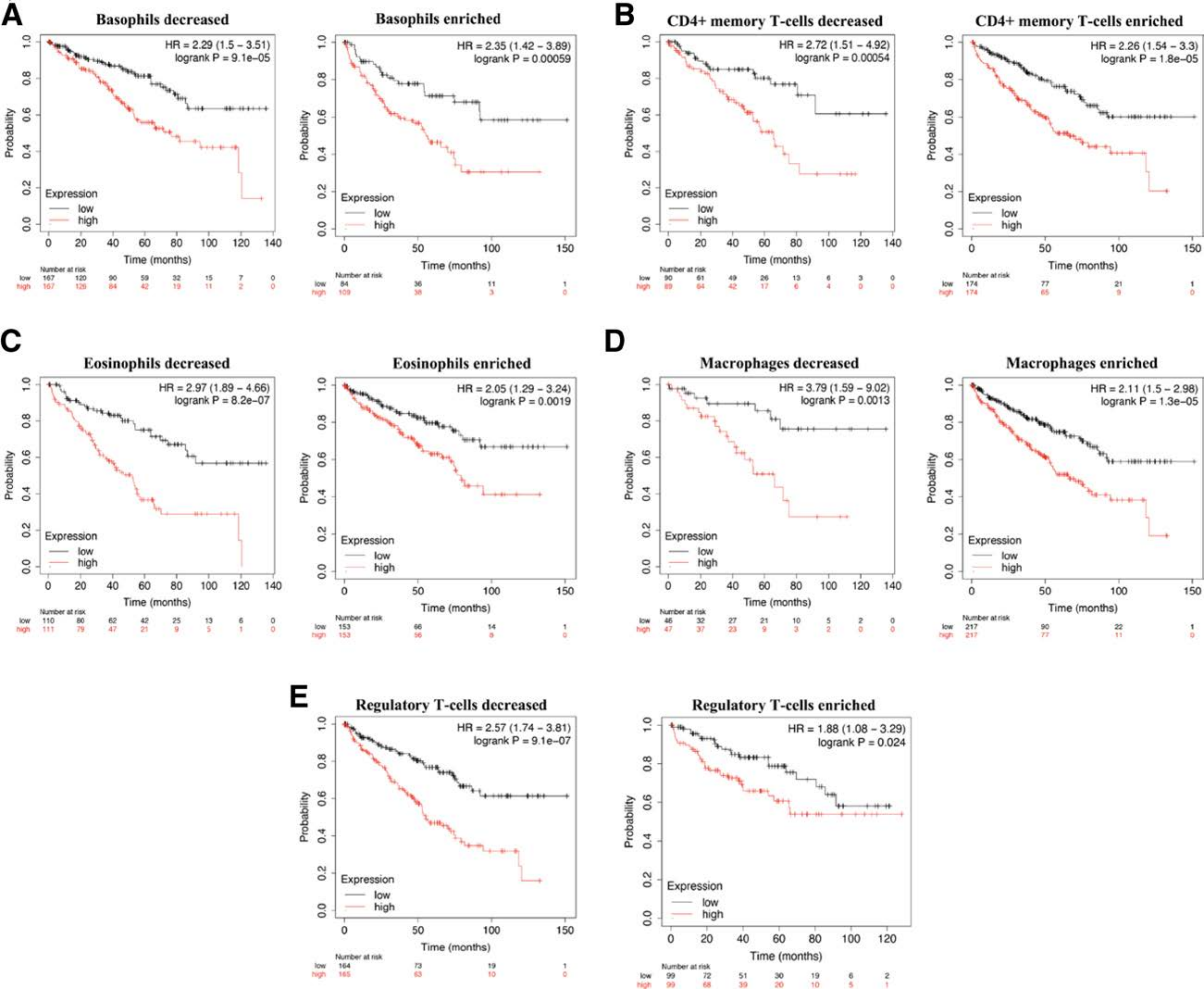
Prognostic value of STAT2 in KIRC based on immune cells subgroups. KIRC = renal clear cell carcinoma.

**Figure 11.**
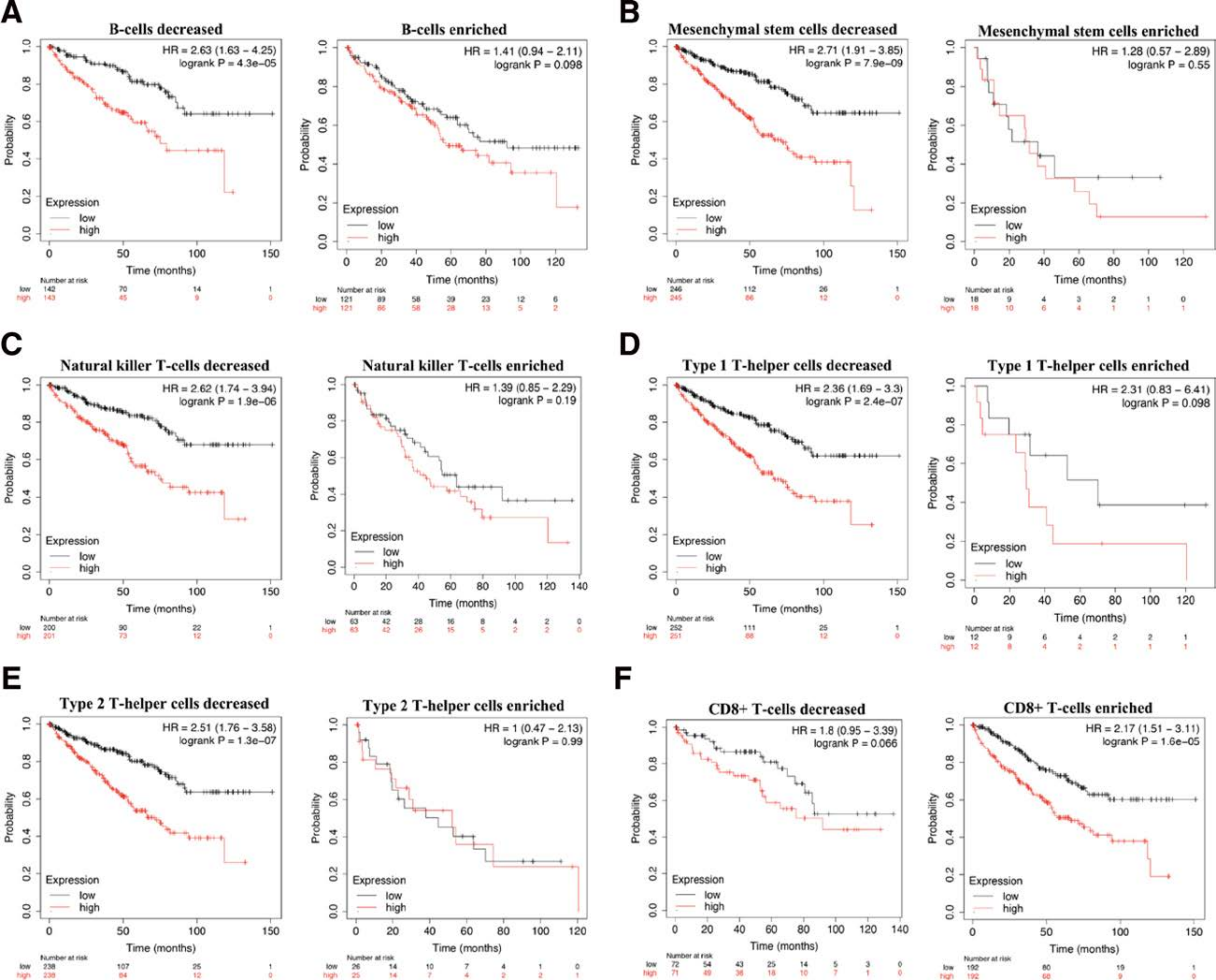
Prognostic value of STAT2 in KIRC based on immune cells subgroups. KIRC = renal clear cell carcinoma.

Above results had indicated that STAT2 was associated with poor prognosis and immune infiltration in KIRC. Previous study also suggested that immune infiltration was correlated with patients’ outcome in KIRC.^[21,22]^ In order to verify whether STAT2 in KIRC could affect the prognosis because of immune infiltration, we next performed prognostic analysis of STAT2 in KIRC based on immune cells. We found that high STAT2 expression in enriched/decreased Basophils cohort (Fig. 10A), enriched/decreased CD4+ memory T-cells cohort (Fig. 10B), enriched/decreased Eosinophils (Fig. 10C), enriched/decreased Macrophages cohort (Fig. 10D), and enriched/decreased regulatory T-cells cohort (Fig. 10E). It’s worth noting that high STAT2 expression of KIRC in decreased B cells cohort (Fig. 11A), decreased Mesenchymal stem cells cohort (Fig. 11B), decreased Natural killer T-cells cohort (Fig. 11C), type 1 T-helper cell cohort (Fig. 11D), and type 2 T-helper cell cohort (Fig. 11E) were associated with poor prognosis (All *P* < .05) while high STAT2 expression of KIRC in enriched CD8+ T cell cohort was associated with a poor prognosis (Fig. 11F). Therefore, STAT2 may affect the prognosis of KIRC patients in part due to immune infiltration.

### 3.7. STAT2 networks of kinase, miRNA, or transcription factor targets in KIRC

**Figure 12.**
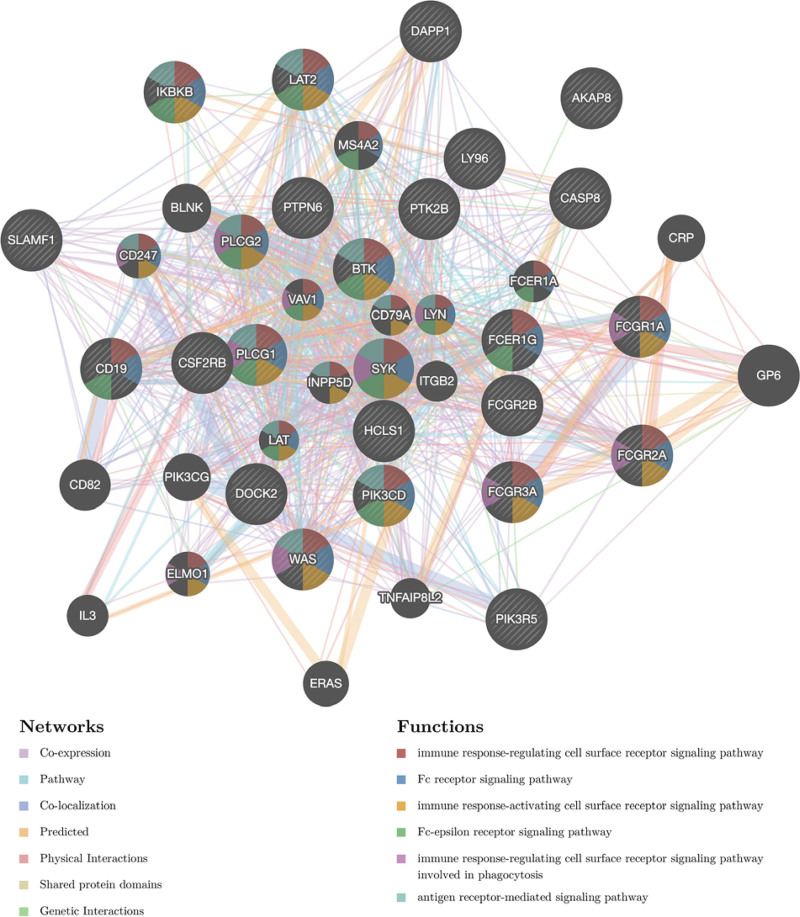
PPI network of kinase_LYN networks (GeneMANIA). PPI network and functional analysis about the gene sets of kinase_LYN networks. The different colors for the network nodes indicate the biological functions of the set of enrichment genes. PPI = protein-protein interaction.

The kinase, miRNA, or transcription factor targets of STAT2 in KIRC were determined using GSEA. LYN, LCK, JAK3, SYK, and HCK were the top 5 most significant kinase targets (Table 4). A PPI network constructed based on the genes correlated with kinase LYN target network revealed that it was enriched in the immune response associated pathway, FC receptor signaling pathway, and antigen receptor-mediated signaling pathway (Fig. 12; Table S1, Supplemental Digital Content, http://links.lww.com/MD/I905). A total of 4 miRNA target networks were significantly involved including MIR-337(GAGCTGG), MIR-296 (GGGGCCC), MIR-518F, MIR-518E & MIR-518A (AGCGCTT), and MIR-191(AGCGCAG) (Table 4). Besides, a PPI network constructed based on the genes correlated with the MIR-337 target network revealed that they were enriched in the antigen receptor-mediated signaling pathway, immune response associated pathway, and the T cell activation and receptor signaling pathway (Figure S2, Supplemental Digital Content, http://links.lww.com/MD/I906, Table S2, Supplemental Digital Content, http://links.lww.com/MD/I907). In the same line, the top 5 most significant transcription factor targets were involved in V$ETS_Q4, V$NERF_Q2, V$NFKAPPAB_01, V$IRF_Q6, and V$STAT3_01 (Table 4). A PPI network constructed based on the genes correlated with transcription factor ETS target network further demonstrated that they were enriched in organelle fusion, single-organism membrane fusion and membrane fusion (Figure S3, Supplemental Digital Content, http://links.lww.com/MD/I908, Table S3, Supplemental Digital Content, http://links.lww.com/MD/I909).

**Table 4:** The Kinase, miRNA and transcription factor-target networks of STAT2 in KIRC (LinkedOmics).

Enriched category	Geneset	LeadingEdgeNum	*P* value
Kinase target	Kinase_LYN	24	0
	Kinase_LCK	22	0
	Kinase_JAK3	7	0
	Kinase_SYK	14	0
	Kinase_HCK	9	.002
miRNA target	GAGCTGG, MIR-337	38	0
	GGGGCCC, MIR-296	23	0
	AGCGCTT, MIR-518F	5	.029
	MIR-518E, MIR-518A	1	.049
	AGCGCAG, MIR-191	3	.048
Transcription factor target	V$ETS_Q4	64	0
	V$NERF_Q2	66	0
	V$NFKAPPAB_01	102	0
	V$IRF_Q6	83	0
	V$STAT3_01	6	.019

KIRC = kidney renal clear cell carcinoma.

## 4. Discussion

STAT proteins are transcription factors that mediate various cellular processes among them differentiation, proliferation, invasion, and apoptosis.^[23]^ Aberrant expression and pleiotropic effects of STATs such as those of STAT2 result in dysregulation of antiviral immunity and tumor pathogenesis.^[5,24]^ Accumulating evidence has highlighted the role of STATs as therapy targets or immune checkpoint inhibitors in various cancers.^[25,26]^ However, the role of STAT2 in KIRC remains controversial. This study therefore investigated various roles of STAT2 protein in KIRC patients.

STAT2 was detected in KIRC patients thereby revealing that it was up-regulated at both the mRNA and protein levels in tumor tissues. Nevertheless, this was not the case in normal healthy persons. Similar results were obtained in subgroup analyses based on sample types, race, gender, age, weight, tumor grade, cancer stage, and nodal metastatic status. Prognostic analysis revealed that patients in the low STAT2 expression group exhibited a better overall survival. These findings demonstrated that STAT2 is a potential prognostic biomarker for KIRC. Actually, previous studies have suggested STAT2 as biomarker for many cancers. In non-small cell lung cancer, STAT2 was a prognostic biomarker and high mRNA expression of STAT2 was significantly associated with shorter overall survival.^[8]^ Moreover, high transcription levels of STAT2 was associated with better relapse-free survival in breast cancer.^[27]^

STATs and the JAK-STAT pathway regulate cytokine associated inflammation and immunity in the tumor microenvironment.^[6]^ Correlation between expression of STAT2, and immune cells, as well as biomarker sets in KIRC patients, revealed that there was a significant positive correlation between expression of STAT2 and immune cells such as B cells, CD8+ T cells, CD4+ T cells, macrophage, Neutrophils, and dendritic cells. Similarly, there was a significant positive correlation between STAT2 expression and immune biomarker sets such as PD-1, CTLA4, and STAT3. These results corroborated with those of Laurence, who reported that there were double-positive CD4+ and CD8+ T Cells as checkpoint inhibitors in renal cell carcinoma (RCC).^[28]^ Elsewhere, Kovaleva reported that RCC-specific macrophages were suitable for therapeutic targeting in RCC.^[29]^ Notably, PD-1 plays a significant role in antitumor activity and has been confirmed to be an immune checkpoint across multiple malignancies.^[30,31]^ On the other hand, CTLA4 is an immune checkpoint for antitumor immunological reactions. It facilitates the Tyk2-STAT3-dependent B-cell oncogenicity.^[32]^ Herein, we found a significant correlation between STAT2, immune cell, and biomarker sets in KIRC. These findings demonstrated that STAT2 is a potential immune checkpoint inhibitor in KIRC.

Furthermore, STAT2 associated genes and their functions in the gene regulation network of KIRC were explored to verify that STAT2 is a potential immune checkpoint inhibitor in KIRC. The functions of STAT2 and its associated genes were primarily implicated in T cell activation, adaptive immune response, cytokine-cytokine receptor interaction, NOD-like receptor signaling pathways, and Toll-like receptor signaling pathway. This was in agreement with the result from a study by Govender who reported that STAT2 could regulate type I Interferon-enhanced IL-10 expression in CD4 T Cells.^[33]^ Of note, the Toll-like receptor is mainly produced by the innate immune cells which significantly mediate and regulate adaptive immune responses. Also, the Toll-like receptor signaling pathway is involved in cancer progression and metastasis.^[34]^ Thus, STAT2 might play an important function in immune escape in the KIRC microenvironment suggesting that STAT2 is a potential immune checkpoint inhibitor for KIRC therapy.

Tumor immune escape and the initiation of metastasis promote oncogenesis and tumor progression.^[35]^ Moreover, the tumor microenvironment is significantly associated with therapeutic response and survival of patients.^[36]^ STAT2 in KIRC was found to be associated with the LYN and LCK kinases network. These kinase networks are involved in the regulation of tumor microenvironment and immune response. Phuong-Hien reported that LYN kinase in the tumor microenvironment exerts an important function in tumor progression of various cancers types.^[36]^ Similarly, a previous study reported that the molecular role of LYN in the control of immunoreceptors can switch to direct homeostasis or inflammation.^[37]^ LCK is an important molecule regulating the functions of T-cells. It is critical in tumor cell signaling and T-cell function.^[38]^ As such, STAT2 might regulate the immune response in the KIRC tumor microenvironment via the LCK and LYN kinase network.

Several miRNAs linked to STAT2 including MIR-337, MIR-296, MIR-518F, MIR-518E, MIR-518A, and MIR-191 were identified. These miRNAs are implicated in the regulation of tumor cell proliferation, invasion, and apoptosis. Moreover, they act as biomarkers in various cancers. Our results were consistent with those of Stefan, who reported that serum miR-191 and miR-337-3 were potential biomarkers in RCC.^[39]^ Similarly, miR-296 was found to suppress tumor metastasis in colorectal cancer via S100A4.^[40]^ At the same time, a previous study reported that miRNA-518 suppressed tumor cell growth and induced apoptosis in gastric cancer.^[41]^ As a consequence, STAT2 can regulate tumor cell proliferation, invasion, and apoptosis via these miRNAs in KIRC.

Nevertheless, this study was limited by several factors. First, the prognostic value, genetic alteration, and associated genes were unverified by a different database such as GEO. As such, future studies should incorporate a verification step to improve the credibility of our findings. Moreover, additional experimental are essential to explore the localization of STAT2 in immune cells.

## 5. Conclusion

In conclusion, our findings reveal that STAT2 was upregulated in KIRC, significantly and positively correlated with immune cells and biomarker sets. Also, it was implicated in immune response, cytokine-cytokine receptor interaction, and Toll-like receptor signaling pathway, indicating that STAT2 is a potential immune checkpoint inhibitor for KIRC therapy.

## Author contributions

**Data curation:** Heng Wang.

Formal analysis: Heng Wang.

Investigation: Tao Zeng, Wen Tian.

Project administration: Jianzhong Ye, Wen Tian.

Software: Heng Wang.

Validation: Jianzhong Ye.

Writing – original draft: Tao Zeng, Wen Tian.

Writing – review & editing: Jianzhong Ye, Wen Tian.

## Supplementary Material












