# Supplementary figures and images for: STAT2 act a prognostic biomarker and associated with immune infiltration in kidney renal clear cell carcinoma
Source: Medicine (Baltimore). 2023 Apr 28;102(17):e33662. doi: 10.1097/MD.0000000000033662 (PMC10146042; doi:10.1097/MD.0000000000033662)

## Slide 1
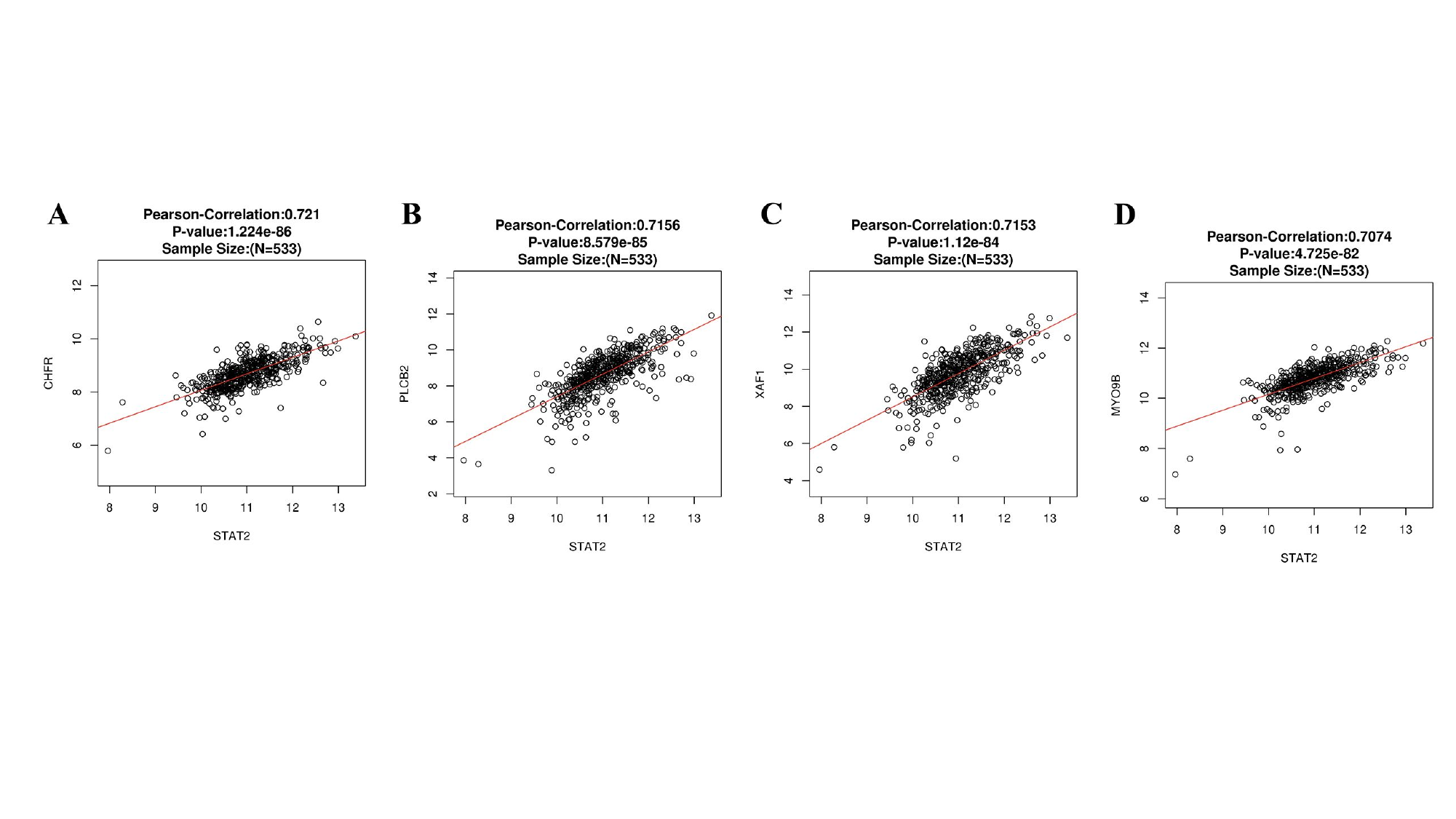

Supplement: Supplementary file 1 [file medi-102-e33662-s001.pptx]

## Slide 1
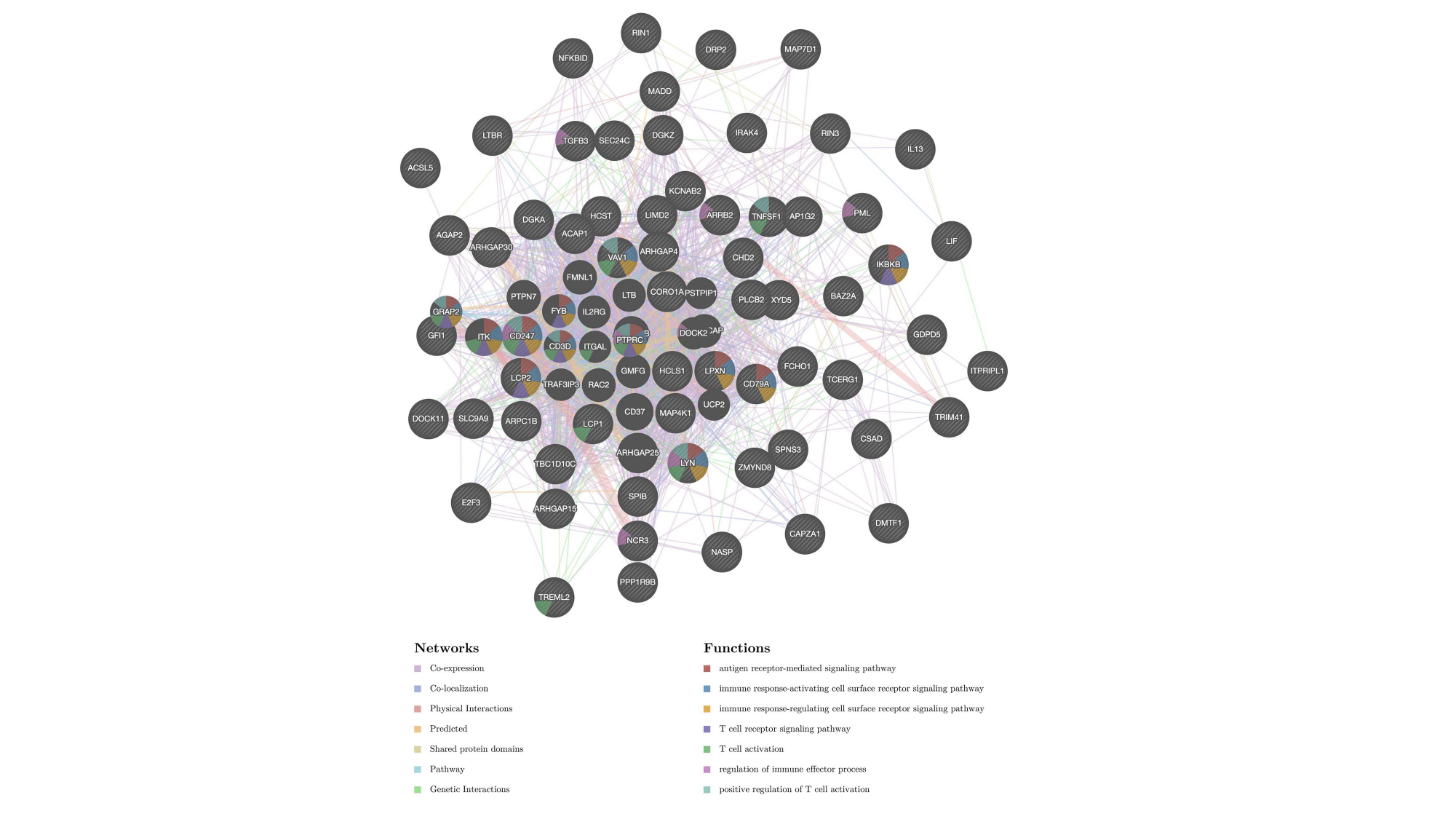

Supplement: Supplementary file 3 [file medi-102-e33662-s003.pptx]

## Slide 1
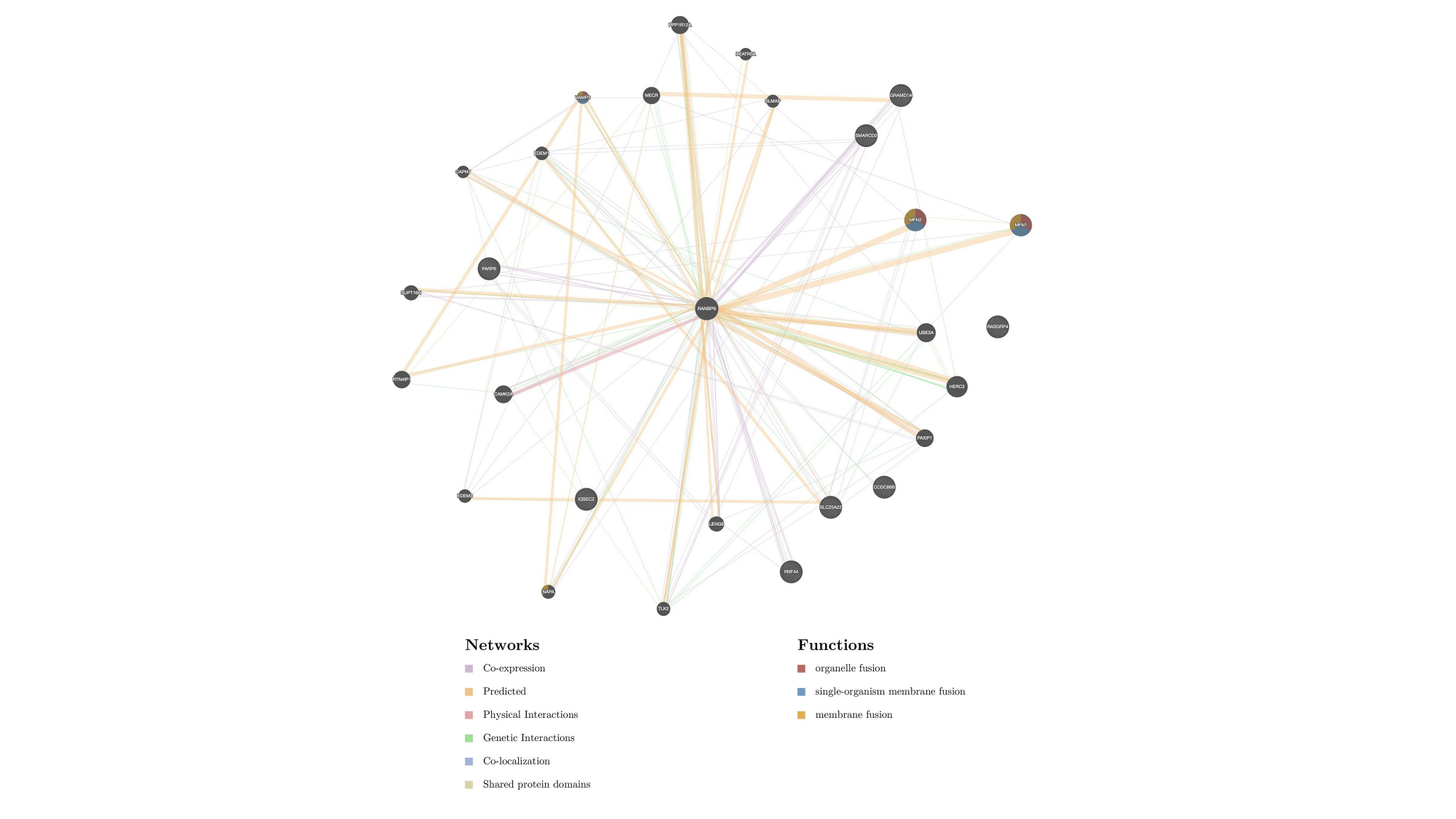

Supplement: Supplementary file 5 [file medi-102-e33662-s005.pptx]
