# Supplementary material for: STAT2 act a prognostic biomarker and associated with immune infiltration in kidney renal clear cell carcinoma
Source: Medicine (Baltimore). 2023 Apr 28;102(17):e33662. doi: 10.1097/MD.0000000000033662 (PMC10146042; doi:10.1097/MD.0000000000033662)
Supplement: Supplementary file 2 [file medi-102-e33662-s002.pdf]

Supplementary Table 1. The LeadingEdgeGene of LYN-Kinase target network (LinkedOmics).

Description	Leading EdgeNum	P-value	LeadingEdgeGene
Kinase_ LYN	24	0	WAS, PTPN6, PIK3CD, SLAMF1, PIK3R5, LAT2, FCGR1A, HCLS1, FCER1G, DOCK2, DAPP1, FCGR3A, IKBKB, BTK, FCGR2C, CD19, PTK2B, PLCG1, CASP8, LY96, AKAP8, FCGR2A, CSF2RB, FCGR2B
