# Supplementary material for: STAT2 act a prognostic biomarker and associated with immune infiltration in kidney renal clear cell carcinoma
Source: Medicine (Baltimore). 2023 Apr 28;102(17):e33662. doi: 10.1097/MD.0000000000033662 (PMC10146042; doi:10.1097/MD.0000000000033662)
Supplement: Supplementary file 4 [file medi-102-e33662-s004.pdf]

Supplementary Table 2. The LeadingEdgeGene of MIR-337 target network (LinkedOmics).

**Description**	**Leading EdgeNum**	**P-value**	**LeadingEdgeGene**
ATGTTAA, MIR-337	38	0	IFFO1, VAMP1, CCDC88B, KCND1, MBD6, MSTO1, ATXN2L, C22orf46, TBC1D2B, ATAD5, LIMK2, PARP6, POGZ, DGKZ, DLEC1, BNIPL, RASGRP4, CAMK2G, IQSEC2, HERC4, EHMT2, 6-Sep, SMARCD2, RNF44, SLC25A22, SEC24C, CD44, ACSL5, NAV1, RANBP10, GRAMD1A, PPP1R9B, ABCA9, SMG1, USP6, TOR2A, CHRD, TXNL4B
