# Supplementary material for: STAT2 act a prognostic biomarker and associated with immune infiltration in kidney renal clear cell carcinoma
Source: Medicine (Baltimore). 2023 Apr 28;102(17):e33662. doi: 10.1097/MD.0000000000033662 (PMC10146042; doi:10.1097/MD.0000000000033662)
Supplement: Supplementary file 6 [file medi-102-e33662-s006.pdf]

Supplementary Table 3. The LeadingEdgeGene of ETS transcription factor target network (LinkedOmics)

Description	Leading EdgeNum	P-value	LeadingEdgeGene
V$ETS_Q4	64	0	PLCB2, FCHO1, MAP4K1, ACAP, DGKA, GFI1, LPXN, PML, VAV1, ARHGAP4, ARHGAP30, TBC1D10C, LIMD2, CSAD, CORO1A, 1-Sep, AGAP2, AP1G2, HCLS1, LCP2, ARPC1B, TCERG1, BAZ2A, IKBKB, KCNAB2, FXYD5, DMTF1, GDPD5, CD247, DGKZ, LIF, RIN1, HCST, RIN3, MAP7D1, LCP1, TRIM41, NASP, E2F3, CAPZA1, ARRB2, CD79A, DRP2, TREML2, SLC9A9, MADD, SPIB, ARHGAP15, SEC24C, SPNS3, ZMYND8, TPRIPL1, LYN, CHD2, ACSL5, LTBR, NCR3, TNFSF11, PPP1R9B, IRAK4, DOCK11, NFKBID, TGFB3, IL13
